# The role of mesenchymal estrogen receptor 1 in mouse uterus in response to estrogen

**DOI:** 10.1038/s41598-023-39474-y

**Published:** 2023-07-29

**Authors:** Keita Furuminato, Saki Minatoya, Eriko Senoo, Tatsuki Goto, Sho Yamazaki, Moeka Sakaguchi, Kenji Toyota, Taisen Iguchi, Shinichi Miyagawa

**Affiliations:** 1 Department of Biological Science and Technology, Faculty of Advanced Engineering, Tokyo University of Science, Niijuku 6-3-1, Katsushika, Tokyo 125-8585 Japan; 2 Noto Marine Laboratory, Institute of Nature and Environmental Technology, Kanazawa University, Noto, Ishikawa 927-0552 Japan; 3 Graduate School of Nanobioscience, Yokohama City University, Yokohama, Kanagawa 236-0027 Japan

**Keywords:** Endocrinology, Reproductive biology

## Abstract

Estrogens play important roles in uterine growth and homeostasis through estrogen receptors (ESR1 and ESR2). To address the role of ESR1-mediated tissue events in the murine uterus, we analyzed mice with a mesenchymal tissue-specific knockout of *Esr1*. *Isl1-*driven *Cre* expression generated *Esr1* deletion in the uterine stroma and endometrium (*Isl-Esr1*KO). We showed that overall structure of the *Isl1-Esr1*KO mouse uterus developed normally, but estrogen responsiveness and subsequent growth were defective, suggesting that mesenchymal ESR1 is necessary for both epithelial and mesenchymal cell proliferation. Furthermore, RNA-seq analysis revealed that the majority of estrogen-induced genes were regulated by stromal ESR1. In control mice, E2 administration induced 9476 up-regulated differentially expressed genes (DEGs), whereas only 1801 up-regulated DEGs were induced by E2 in *Isl1-Esr1*KO mice. We further showed that stromal ESR1-regulated genes in the mouse uterus included several growth factors and cytokines, which are potential factors that regulate epithelial and stromal tissue interaction, and also genes involved in lipid homeostasis. Therefore, we infer that stromal ESR1 expression is indispensable for most estrogen actions in the mouse uterus and the current results provide new insights into estrogen-mediated homeostasis in female reproductive organs.

## Introduction

Estrogens play important roles in vertebrate reproductive biology and their biological effects are principally mediated through estrogen receptors (ESRs), which are ligand-dependent transcription factors. Two ESRs (ESR1/ERα and ESR2/ERβ) have been identified from a variety of vertebrate species and the actions of estrogens on the uterus have been analyzed extensively. The uterus is comprised primarily of an epithelium, mesenchyme-derived stroma and smooth muscle and all of these tissues express ESR1. ESR1 is the predominant ESR subtype mediating the proliferative and differentiative effects of estrogens. Administration of estrogens increases uterine weight and promotes cell proliferation and differentiation and these effects were ablated in *Esr1* KO mice^1,2^.

Epithelial-stromal interactions play pivotal roles in organogenesis, tissue differentiation and homeostasis in variety of organs. In the uterus, epithelial-stromal interactions have been demonstrated using ex vivo tissue recombination experiments with *Esr1* KO- and wild type-derived epithelium and stroma. According to such experiments, effects of estrogens on uterine epithelial cell proliferation are mediated primarily via stromally-expressed ESR1^3^. Thus, estrogen-induced growth factors secreted from the stroma were postulated to promote epithelial cell proliferation in a paracrine manner. IGF1 is a potential paracrine mediator for estrogen-induced uterine epithelial cell proliferation. Estrogen administration induced IGF1 expressed in the stroma and activated IGF1-IGF receptor signal in the epithelium^4,5^; however, IGF1 treatment alone cannot substitute for estrogen action^6,7^. In addition to mitogenic effects, the expression of various genes is regulated by paracrine signaling. Progesterone receptor (PGR) is expressed in the epithelial cells but not in the stromal cells in the absence of estrogen stimulation, whereas estrogen stimulation shifted PGR expression, decreasing epithelial PGR expression and up-regulated PGR expression in the stroma. The regulation of steroid hormone receptor expression and the subsequent control of cessation and activation of cell proliferation is required for pregnancy. Inhibition of epithelial PGR expression by estrogens does not occur in the absence of stromal ESR1 in tissue recombination experiments^8^. Epithelial ESR1 is dispensable for these events, although epithelial ESR1 expression is required for epithelial functionalization, such as lactoferrin (*Ltf*) gene expression and protein secretion^9^.

Subsequent in vivo mouse analyses supported such epithelial-stromal interactions observed in tissue recombination experiments^10,11^. Experiments using epithelial cell-specific KO of *Esr1* driven by *Wnt7a-Cre* mouse line revealed that estrogen could induce proliferation of uterine epithelial cells despite the absence of epithelial ESR1^10^. Similar approaches were conducted using an anti-Müllerian hormone receptor (*Amhr*)*-Cre* mouse line to investigate stromal ESR1 function^11^. In this mouse model, expression of ESR1 remained intact in the mesometrial side whereas *Amhr*-driven ablation of *Esr1* was limited to the stromal cells of the anti-mesometrial side, with variable degrees of *Esr1*-deletion in individual animals. This study demonstrated that proliferative activity was reduced in the epithelial cells adjacent to stromal cells in which ESR1 was not expressed. However, due to a limited number of KO cells, the functional requirements of stromal ESR1 during estrogen-induced events in the uterus are not fully understood.

Epithelial-stromal interactions mediating estrogen action are commonly observed in other organs including mammary gland, oviduct, and vagina, and epithelial ESR1 functions in each organ were reported^12–14^. Until now, no mouse model for complete KO of uterine stromal Esr1 has been developed. Here we generated whole stromal ESR1-deletion in the mouse uterus by crossing an *Isl1-Cre* mouse line with *Esr1*-floxed mice to provide new insights into potential tissue-specific function of ESR1. We demonstrated that deletion of stromal ESR1 in the mouse uterus resulted in overall loss of estrogen action, including epithelial cell proliferation. Taking advantage of this in vivo mouse model, we evaluated gene expression by RNA-seq, and showed a large reduction in the number of estrogen-induced genes. Thus, stromal ESR1 controls a majority of the estrogen response and is indispensable for homeostasis in mouse uterus.

## Results

### Phenotypes of uterine mesenchyme-specific Esr1 Knockout Mice

**Figure 1:**
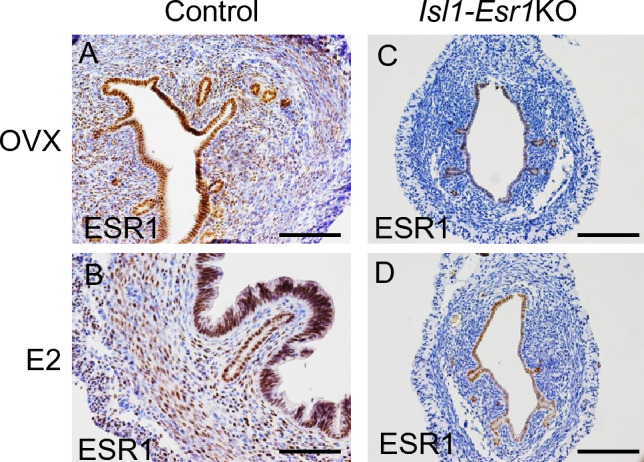
Stomal cell-specific ESR1 deletion in the *Isl1-Esr1*KO mice. ESR1 protein expression is evaluated by immunohistochemistry in control (**A**, **B**) and *Isl1-Esr1*KO (**C**, **D**) mice treated with OVX only (**A**, **C**) or E2 for three consecutive days (**B**, **D**), indicating successful deletion of *Esr1* specifically in the stromal cells in the *Isl1-Esr1*KO mice. Scale bars, 100 μm.

ESR1 protein is expressed in all epithelial cells, most stromal and smooth muscle cells in the wild-type uterus (Fig. 1A,B). We generated a uterine stromal cell-specific *Esr1* knockout mouse model (*Isl1-Esr1*KO) by crossing *Esr1*-floxed mice with *Isl1-Cre* knockin lines expressing Cre recombinase in caudal mesenchymal tissues^15^. ESR1 protein was detected in the epithelium but not in the stroma or smooth muscles (myometrium), demonstrating the mesenchyme-specificity of ESR1 loss-of-function in the *Isl1-Esr1*KO mouse uterus (Figs. 1C,D, and S1). Uterine stromal ESR1 has been lost at the neonatal stage in the *Isl1-Esr1*KO mice (Fig. S3). We note that *Isl1-Cre* is also active in a broad range of tissues^16,17^, and other tissues might be affected but were not studied here. Female reproductive tracts in the *Isl1-Esr1*KO mice were hypoplastic and ovaries lacked corpora lutea at 8 weeks of age. Histologically, the ovaries had few mature follicles and hemorrhagic cysts (Fig. S3), which resembles the ovary of conventional *Esr1*KO mice^1,2^. Thus, in the current study, we investigated histology and gene expression in the *Isl1-Esr1*KO mouse uterus using ovariectomized (OVX) mice to avoid any confounding effects of hypothalamus-pituitary–gonadal axis, and to simplify analysis of hormonal effects.

**Figure 2:**
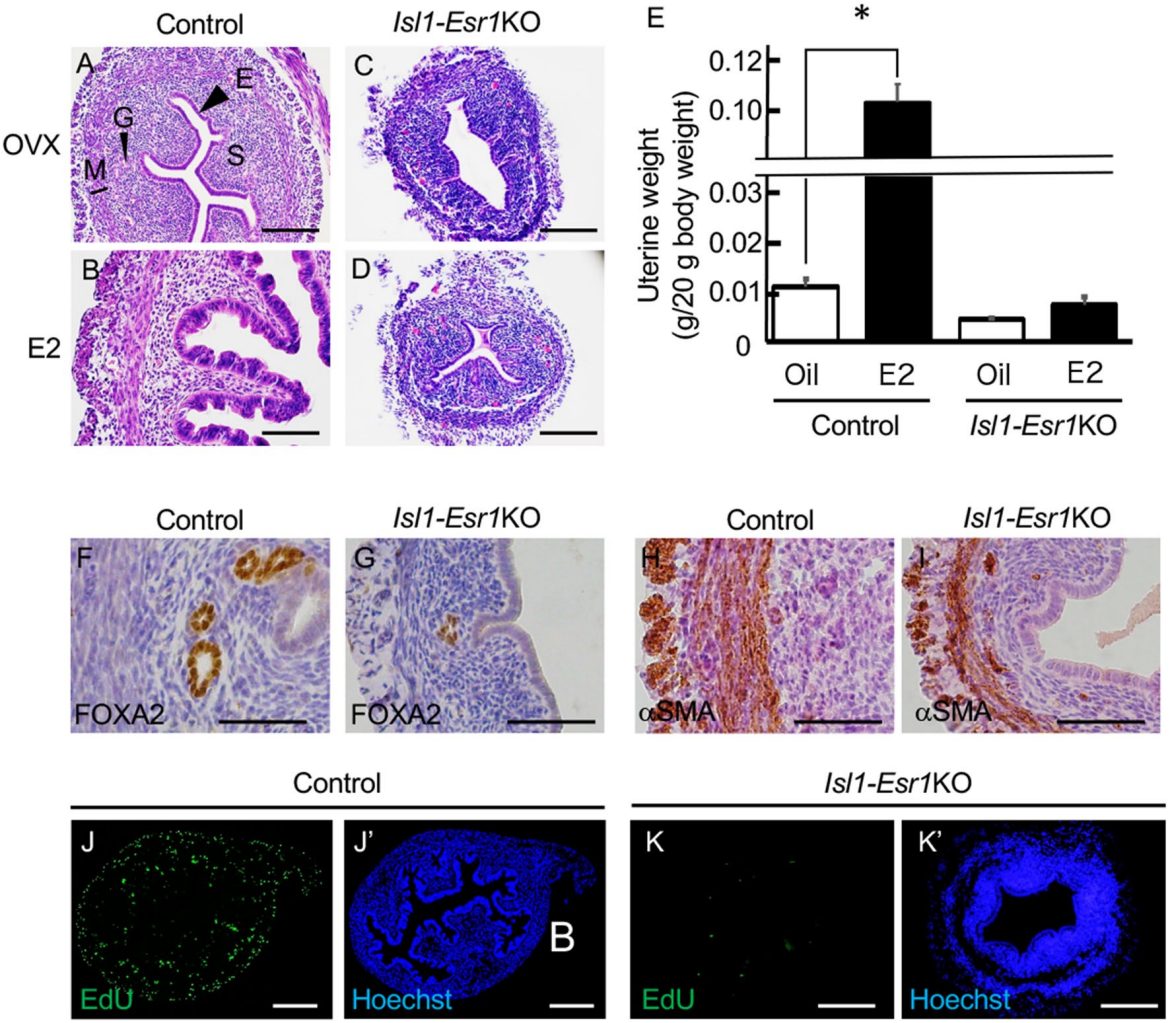
Effects of stomal cell-specific ESR1 deletion in mouse uterus. The uterus of OVX control (**A**) and *Isl1-Esr1*KO (**C**) mice exhibit hypoplastic phenotypes. E2 treatment induces uterine organ growth and epithelial cell hyperplasia in the control (**B**), but fails to such phenotypes in the uterus of *Isl1-Esr1*KO mice (**D**). Uterine organ weight increases by E2 in controls, but not changed in *Isl1-Esr1*KO mice (**E**). More than 5 animals were analyzed. Error bars represent SEM. * indicates significant difference compared with OVX group assessed by student’s t-test (*p* < 0.05). Expression pattern of marker proteins FOXA2 for uterine gland (**F**, **G**) and αSMA for smooth muscle (**H**, **I**) in 8-week-old OVX mice uterus. Even in the absence of stromal ESR1, uterine glands are developed and smooth muscle cells ware differentiated. EdU-incorporation is detected in the control (**J**) and *Isl1-Esr1*KO (K) mouse uterus treated with E2 for three consecutive days, and representative images are shown. Blue fluorescent signal (**J**’, **K**’) indicates Hoechst staining in the same image of EdU staining (**J**, **K**). Scale bars, 200 µm (**J**), 100 μm (**A**–**D**, **K**) or 50 µm (**F**-**I**).

The uterus of 8-week-old control OVX mice was composed of a single layer of low columnar epithelial cells with relatively involuted stroma (Fig. 2A), and 17β-estradiol (E2) administration induced epithelial hypertrophy with water imbibition (Fig. 2B). The uterus of *Isl1-Esr1*KO OVX mice possess the definitive compartments, the epithelium, stroma and myometrium, however, stroma was less organized and hypotrophic compared with that of the controls (Figs. 2C and S1). Whole uterine weights were approximately half between control and *Isl1-Esr1*KO OVX mice in oil control injections (Fig. 2E). E2 administration for three consecutive days induced 10-folds increase in uterine wet weight in controls, but has no significant effects on uterine growth and weight in *Isl1-Esr1*KO mice (Figs. 2D,E, S1, and S4A). Luminal and glandular epithelial cells appeared normal but consistently low columnar morphology and lacked tall columnar structure in the E2-treated *Isl1-Esr1*KO mouse uterus. *Isl1-Esr1*KO uterus expressed Forkhead box A2 (FOXA2), an uterine gland marker, but a sparse distribution of uterine glands compared with those of controls (Figs. 2F,G, and S4B). Alpha-smooth muscle actin (αSMA) was normally expressed, suggesting normal differentiation of muscle tissue, but was somewhat disorganized (Fig. 2H,I). Thus, overall structure of *Isl1-Esr1*KO mouse uterus was reasonably normal but estrogen responsiveness and subsequent growth were impaired.

### Epithelial-stromal tissue interaction in mouse uterus

Ex vivo tissue recombination experiments demonstrated that stromal ESR1 is required for paracrine regulation of epithelial cell proliferation and expression of several genes^3,8^. Incorporation of the deoxy-thymidine analog 5-ethynyl-2-deoxyuridine (EdU) showed that E2 administration induced cell proliferation in both epithelial and stromal cells in control mice (Fig. 2J). Note that stomal cell proliferation was not restricted in ESR1-expressing cells (Fig. S5). By contrast, proliferation of stromal and luminal epithelial cells of *Isl1-Esr1*KO mice uterus were not increased even after E2 administration (Fig. 2K). Accordingly, cyclin-dependent kinase inhibitor 1A (*Cdkn1a*) gene expression, a cell proliferation marker, was not induced by E2 administration in the *Isl1-Esr1*KO mice uterus (Fig. S6).

**Figure 3:**
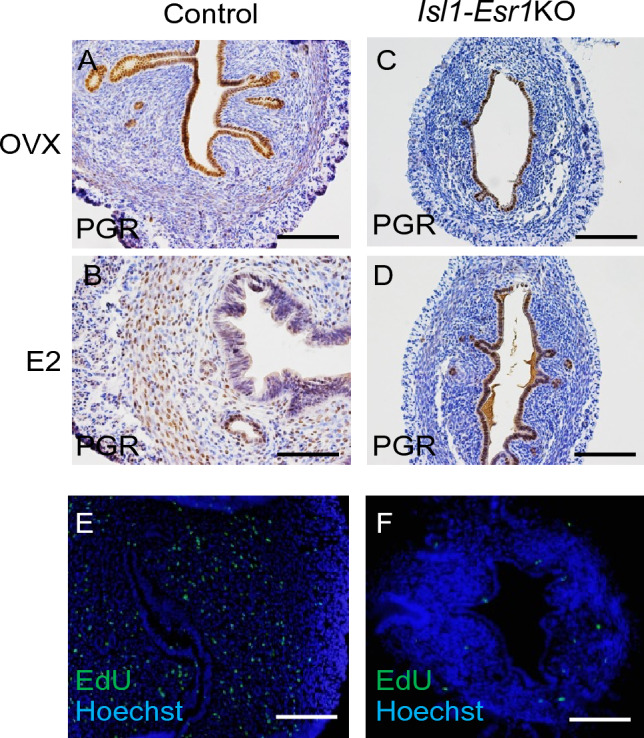
Regulation of PGR expression by in mouse uterus. Immunohistochemistry of PGR in control (**A**, **B**) and *Isl1-Esr1*KO (C) mice treated with OVX only (**A**, **C**) or E2 (**B**, **D**). PGR expression is detected in uterine epithelium (**A**), but E2 administration downregulates PGR expression in the epithelium, while upregulated in stroma (**B**). The shift of PGR expression is not observed in the *Isl1-Esr1*KO mice (**C**, **D**). Proliferation of the uterine stromal cells after a series of E2 and P4 treatments is detected EdU-incorporation (**E**, **F**). *Isl1-Esr1*KO mouse uterus fails to increase of stromal cell proliferation (**F**). Blue fluorescent signal indicates Hoechst staining. Scale bars, 100 μm.

The uteri of OVX control and *Isl1-Esr1*KO mice expressed PGR in the epithelial cells but not in the stromal cells (Fig. 3A,C). Upon E2 administration, epithelial PGR was downregulated whereas stromal PGR was upregulated in controls (Fig. 3B). However, the *Isl1-Esr1*KO mouse uterus consistently expressed PGR in the epithelial cells but not in the stromal cells (Fig. 3D), suggesting that downregulation of PGR in the epithelial cells depends on stromal ESR1, and PGR expression in stromal cell depends on stromal ESR1.

During embryo implantation in normal mice, estrogen and progesterone cooperatively regulate the cessation of uterine epithelial cell proliferation and subsequent stromal cell proliferation^18^. We used hormonal regimens to mimic the hormonal profile during embryo implantation, in which 3 days of progesterone (P4) injection permits the uterine luminal epithelia to differentiate into a pre-receptive state, and the combination of P4 and E2 on the fourth day induced the receptive state^19^. In control mice, cell proliferation in the epithelial cells was not observed but was augmented in the stromal cells (Fig. 3E). However, in *Isl1-Esr1*KO mice, both epithelial and stromal cell proliferation was decreased (Fig. 3F).

### Evaluation of E2-specific responses in mouse uterus

**Figure 4:**
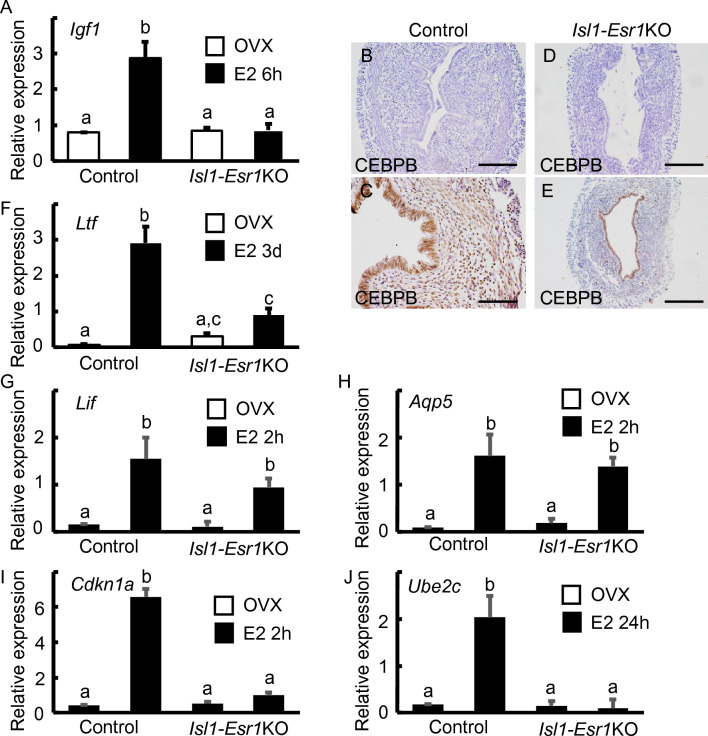
Expression patterns of estrogen-regulated genes in the mouse uterus. *Igf1* gene expression at 6 h after E2 administration is induced in control uteri, but not changed in the *Isl1-Esr1*KO mouse uteri (**A**). CEBPB protein is induced in both epithelial and stromal cells (**B**, **C**), but only epithelial cells express CEBPB after E2 administration in the *Isl1-Esr1*KO mouse uterus (**D**, **E**). Control mouse uteri increased *Ltf* gene expression but lost some estrogen response in the *Isl1-Esr1*KO mouse uterus (**F**). *Lif* (**G**) and *Aqp5* (**H**) genes expression is increased in both E2 treated control and* Isl1-Esr1*KO mouse uterus. *Isl1-Esr1*KO mouse uterus. Loss of *Cdkn1a* (**I**) and *ube2c* (**J**) genes expression in the E2 treated *Isl1-Esr1*KO mouse uterus. Results are mean ± SEM. A two-way ANOVA followed by a Tukey–Kramer test was used and *p* < 0.05 was considered as significantly different.

We next investigated the expression of representative estrogen-regulated genes involved in cell proliferation such as *Igf1* and CCAAT/enhancer-binding protein β (*Cebpb)*^10,20^. *Igf1* is a stromally expressed gene and E2 administration significantly upregulated its expression at 6 h in controls (Fig. 4A). By contrast, the expression levels of *Igf1* in *Isl1-Esr1*KO mouse uterus were not significantly different (Fig. 4A). Expression of CEBPB protein was induced by E2 administration in the epithelial and stromal cells of control mouse uteri (Fig. 4B,C). Epithelial CEBPB immunoreactivity was found in the epithelium but not in the stroma of *Isl1-Esr1*KO uteri (Fig. 4D,E).

LTF is a secreted protein regulated by E2 in mammalian uterine epithelium^10^. Control mouse uteri increased *Ltf* gene expression but lost some estrogen response in the *Isl1-Esr1*KO mouse (Fig. 4F), suggesting that full *Ltf* expression is required for both epithelial and stromal ESR1 as previously reported^9^. Remarkably, *Ltf* expression was augmented in the *Isl1-Esr1*KO mice compared to controls in the absence of estrogen. Other estrogen-regulated genes were also evaluated. Expression of early estrogen responsive genes, leukemia inhibitory factor (*Lif*), *Cdkn1a*, and aquaporin (*Aqp5*) was upregulated at 2 h after E2 administration in the control mice uterus (Fig. 4G–I). Of those, *Lif* and *Aqp5* expression were similarly increased in the *Isl1-Esr1*KO mouse uterus, but *Cdkn1a* was not (Fig. 4G–I). The late estrogen responsive gene, ubiquitin-conjugating enzyme E2C (*Ube2c*) was not induced at 24 h in the *Isl1-Esr1*KO mice (Fig. 4J).

### Gene expression analysis by RNA-seq

**Figure 5:**
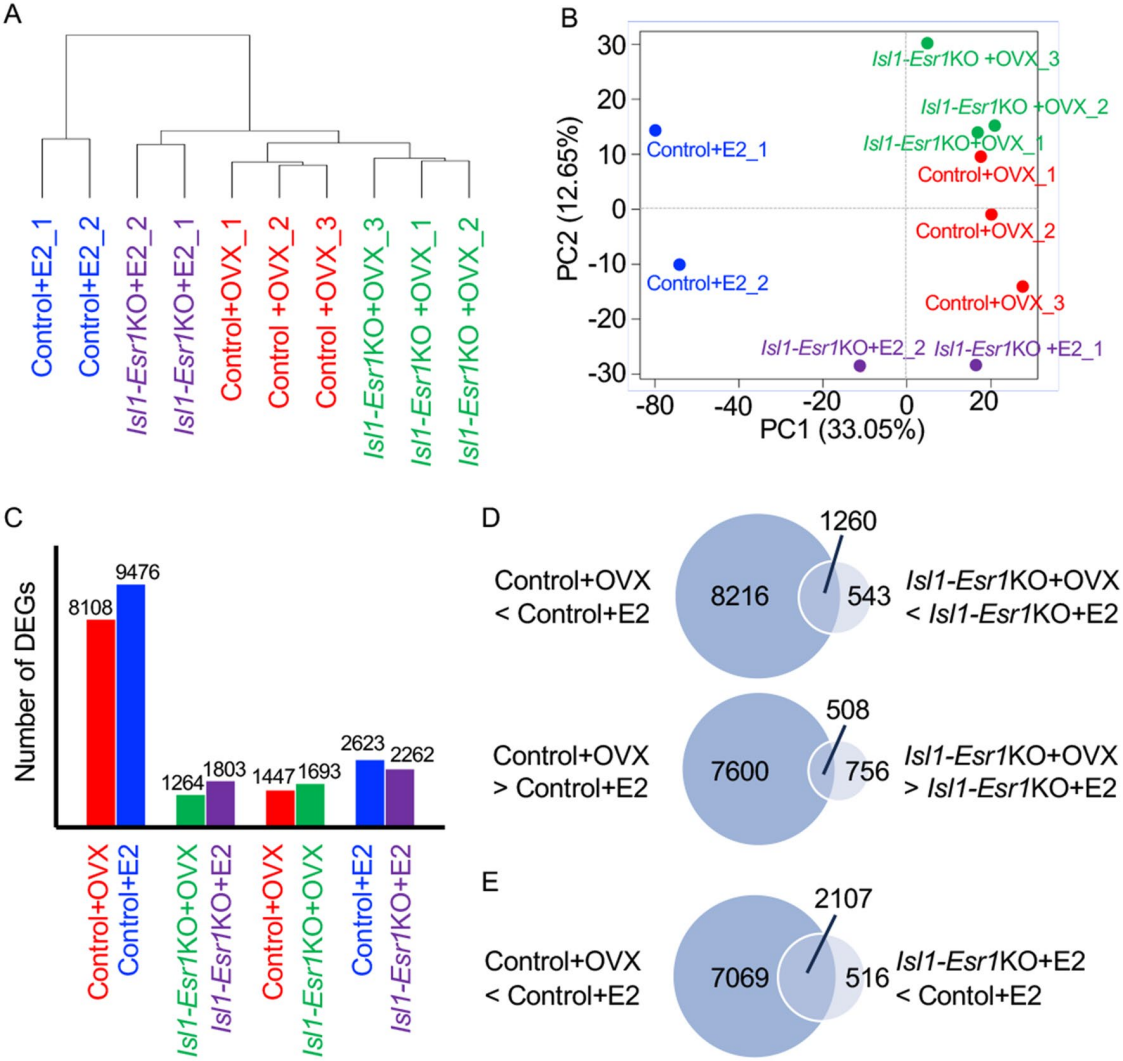
RNA-seq analysis of uterine transcripts from control and *Isl1-Esr1*KO mice treated with vehicle and E2 at 6 h. Cluster dendrogram (**A**) and principal component analysis (PCA) plot showing the first and second principal components of all replicants (**B**) shows control E2 group (CON + E2) is differentially clustered from other group. Number of differentially expressed genes (DEGs) among uteri from control and *Isl-Esr1*KO mice treated with vehicle (Control + OVX or *Isl1-Esr1*KO + OVX) or E2 (Control + E2 or *Isl1-Esr1*KO + E2) at 6 h (**C**). Venn diagram of number of DEGs by E2 administration between control and *Isl1-Esr1*KO mouse uterus (**D**), and genes implicating stromal ESR1-induced genes (**E**).

The response of the OVX mouse uterus to E2 was apparent within 6 h. These included metabolic responses in the form of increased water imbibition, vascular permeability and hyperemia, prostaglandin release, glucose metabolism, eosinophil infiltration, RNA polymerase and chromatin activity, lipid and protein synthesis^21,22^. Thus, we conducted RNA-seq analysis of uteri from control and *Isl-Esr1*KO mice treated with vehicle (Control + OVX or *Isl1-Esr1*KO + OVX) or E2 (Control + E or *Isl1-Esr1*KO + E) at 6 h (Fig. 5).

The cluster dendrogram and principal component analysis (PCA) of all replicates showed that the Control + E2 group is differentially clustered from other groups (Fig. 5A,B). In control mice, E2 administration induced 9476 up-regulated differentially expressed genes (DEGs) and 8108 down-regulated DEGs, whereas, in *Isl1-Esr1*KO mouse, 1803 up-regulated DEGs and 1264 down-regulated DEGs (Fig. 5C; DEGs are given in Supplemental Tables S2-S5). Thus, approximately 90% of estrogen-induced genes were regulated by stromal ESR1 (Fig. 5D). We found 1447 Control + OVX biased genes and 1693 *Isl1-Esr1*KO + OVX biased genes between Control + OVX and *Isl1-Esr1*KO + OVX groups (Fig. 5C), indicating that the *Isl1-Esr1KO* mouse uterus was strongly affected by loss of stromal ESR1 function even in the absence of E2. When comparing E2-administered control and *Isl1-Esr1*KO mouse uterus, the number of DEGs are increased, and 2623 DEGs were control-E2 biased while 2262 DEGs were *Isl1-Esr1*KO-E2 biased (Fig. 5C).

The genes commonly found as DEGs between Control + OVX and Control + E2, and between *Isl1-Esr1*KO + OVX and *Isl1-Esr1*KO + E2 were postulated as epithelial expressed ESR1-madiating genes. We found 1260 up-regulated and 508 down-regulated genes were identified, which were satisfied such criteria (Fig. 5D and Supplement Table S6). Gene ontology (GO) analysis for biological process revealed that the ribosome biology and RNA processing are enriched (Supplement Table S7).

**Figure 6:**
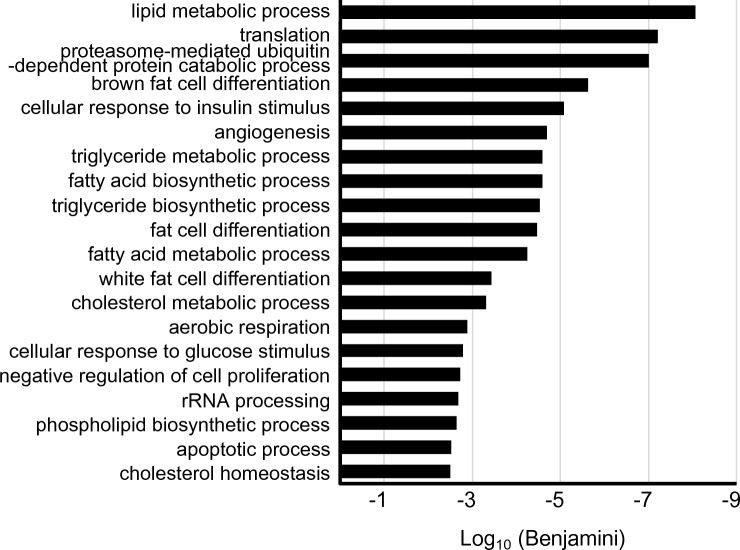
Top 20 biological process gene ontology (GO) terms mapped to the candidate stromal ESR1-mediated events in mouse uterus. The terms related lipid, triglyceride, and fatty acid metabolism, and fat cell differentiation are enriched.

We next focused on estrogen-induced genes in the stromal cells. We investigated the upregulated genes by E2 administration in controls (Control + OVX < Control + E2) and the augmented genes in control E2 uterus rather than those of *Isl1-Esr1*KO E2 uterus (*Isl1-Esr1*KO + E2 < Control + E2). We identified 2107 genes that satisfied this criterion (Fig. 5E and Supplement Table S8). Expression of these genes was not induced without stromal ESR1. Secreted growth factors that could be candidates for mediating epithelial and stromal tissue interaction were identified. These included *Igf1* and several Wnt ligands (*Wnt4*, *Wnt7b*, *Wnt9a*, *Wnt9b*), fibroblast growth factors (*Fgf1*, *Fgf21*), neuregulins (*Nrg2*, *Nrg4*), transforming growth factor beta (Tgf-β) superfamily member [*Tgfb2*, bone morphogenetic proteins (*Bmp1*, *Bmp6*), growth differentiation factors (*Gdf6*, *Gdf15*), inhibin beta-B (*Inhbb*)], and other cytokines such as chemokine (C–C motif) ligands (*Cxcl6*, *Cxcl7*, *Cxcl9*, *Cxcl11*, *Cxcl12*), interleukin (*Il11*), vascular endothelial growth factor member [*Vegfa*, placental growth factor (*Pgf*)] (Table 1). Further, GO analysis for biological process revealed that terms related to lipid, triglyceride, fatty acid metabolism, and fat cell differentiation were enriched (Fig. 6 and Supplement Table S9). These included important lipid metabolism regulatory genes such as *Cebpa*, *Cebpb*, sterol regulatory element binding transcription factor 1 (*Srebf*1), Kruppel-like factor 4 (*Klf4*), activating transcription factor 5(*Atf5*) (Table 2).

**Table 1:** Expression profiles for selected secreted growth factor and cytokine genes.

Id	Gene	log2 FoldChange Control + OVX < Control + E	padj	log2 FoldChange Control + E > *Isl1-Esr1*KO + E	padj
ENSMUST00000115713	*Nrg2*	3.124	1.93E−57	− 3.058	8.46E−22
ENSMUST00000108783	*Wnt9a*	2.165	1.61E−52	− 2.612	4.15E−16
ENSMUST00000004913	*Pgf*	4.955	6.91E−40	− 3.948	3.85E−53
ENSMUST00000095360	*Igf1*	3.082	1.51E−34	− 3.31	4.83E−24
ENSMUST00000071648	*Vegfa*	2.32	9.77E−32	− 2.162	7.89E−07
ENSMUST00000110103	*Gdf15*	8.042	1.93E−31	− 4.036	8.42E−05
ENSMUST00000073043	*Cxcl12*	1.564	2.32E−29	− 1.279	4.26E−07
ENSMUST00000045747	*Wnt4*	2.968	4.11E−27	− 2.882	7.88E−06
ENSMUST00000038765	*Inhbb*	4.596	2.18E−22	− 4.738	2.32E−18
ENSMUST00000040750	*Lif*	4.271	2.29E−20	− 2.731	0.0307
ENSMUST00000109424	*Wnt7b*	3.556	5.53E−14	− 2.334	0.0090
ENSMUST00000019266	*Cxcl9*	2.489	2.53E−10	− 2.162	0.0002
ENSMUST00000019071	*Cxcl6*	2.853	9.22E−10	− 2.401	0.0000
ENSMUST00000033099	*Fgf21*	8.565	1.40E−09	− 7.836	8.05E−05
ENSMUST00000000194	*Cxcl12*	4.058	1.61E−09	− 2.481	0.0029
ENSMUST00000021011	*Cxcl7*	4.799	6.80E−09	− 3.361	0.0066
ENSMUST00000045288	*Tgfb2*	1.867	3.14E−06	− 1.446	0.0015
ENSMUST00000171970	*Bmp6*	2.348	9.59E−05	− 2.259	0.0165
ENSMUST00000094892	*Il11*	3.802	0.0002	− 3.092	0.0039
ENSMUST00000057613	*Gdf6*	3.181	0.0003	− 3.957	0.0070
ENSMUST00000000342	*Cxcl11*	1.699	0.0005	− 1.166	0.0214
ENSMUST00000040647	*Fgf1*	0.81	0.0058	− 1.259	0.0442
ENSMUST00000018630	*Wnt9b*	2.422	0.0102	− 5.767	0.0439
ENSMUST00000126368	*Nrg4*	4.135	0.0346	− 7.795	0.0159
ENSMUST00000022693	*Bmp1*	0.464	0.0390	− 0.858	0.0406

**Table 2:** Expression profiles for selected transcriptional factor for regulating lipid metabolism.

Id	Gene	log2 FoldChange Control + OVX < Control + E	padj	log2 FoldChange Control + E > *Isl1*− *Esr1*KO + E	padj
ENSMUST00000070642	*Cebpb*	2.591	5.26E−96	1.765	3.32E−09
ENSMUST00000047356	*Atf5*	2.653	2.85E−47	1.989	4.67E−08
ENSMUST00000042985	*Cebp1*	4.089	2.64E−15	3.086	2.82E−06
ENSMUST00000107619	*Klf4*	2.716	2.25E−14	1.745	5.00E−05
ENSMUST00000020846	*Srebf1*	1.65	3.24E−10	1.339	0.00286
ENSMUST00000171644	*Pparg*	3.871	2.09E−07	3.259	8.26E−05

## Discussion

Epithelial-stromal interactions are essential for regulating organogenesis, tissue/cell differentiation and functions throughout the body. Cell proliferation and differentiation in female reproductive organs have been studied extensively as an excellent model to analyze such tissue interactions. Tissue recombination experiments^23^ suggested that epithelial cell proliferation in female reproductive organs, including uterus, vagina and mammary gland, is mediated by stromal ESR1 in a paracrine manner^3,24,25^. Therefore, epithelial ESR1 may be dispensable for epithelial mitogenic response to estrogens. Subsequent genetic studies using an epithelial cell-specific *Esr1*KO mouse model demonstrated that epithelial ESR1 is neither necessary nor sufficient for uterine cell proliferation in female reproductive organs^10,13,26^. Winuthayanon et al*.*^11^ reported that epithelial cells failed to proliferate without ESR1 in neighboring stromal cells. However, the function of ESR1 has not been fully investigated, because of a lack of efficient Cre mouse lines for stromal cell-specific KO of *Esr1*. To clarify and extend those observations, we used an *Isl1-Cre* mouse line and *Esr1*-floxed mouse, to create stromal-specific knockout of ESR1, which allowed us to investigate the roles of stromal ESR1 in mediating the effects of E2 in the mouse uterus. We found that stromal ESR1 is necessary for epithelial cell proliferation, which is consistent with the previous tissue recombination experiments, but our findings also demonstrated that stromal ESR1 is indispensable for organ growth and for the majority of estrogen-induced actions in mouse uterus.

### Phenotypes and response to E2 in the Isl1-Esr1KO mouse uterus

The uterus is derived from the Müllerian duct and consists of an epithelium and mesenchyme during early development. During neonatal development, the mesenchyme further differentiates into stoma and smooth muscle cells (an outer longitudinal and an inner circular smooth muscle layer). Previous reports using a conventional *Esr1*KO mouse line showed that ESR1 is not required for uterine tissue differentiation^1,2^. Similarly, *Isl1-Esr1*KO mouse uterus did not response to E2 for cell proliferation in both epithelium and stroma, resulting in hypoplastic phenotypes.

It is postulated that a stroma-derived secreted growth factor mediates estrogen-induced uterine epithelial cell proliferation in a paracrine manner. Several growth factors were proposed to fill this role and IGF1 is considered as a plausible candidate; *Igf1* is expressed predominantly in the stroma upon estrogen stimulation, accompanied by phosphorylation pf IGF1 receptor in the epithelium^5,27^. Moreover, IGF1 administration can elicit epithelial cell proliferation in vivo^6,10,28^. By contrast, tissue grafting experiments using *Igf1* KO mouse uteri showed that systemic but not local IGF1 is required for E2-induced uterine epithelial cell proliferation^7^. Thus, complementary or combination of other growth factors will be required for paracrine induction of uterine epithelial cell proliferation.

In the current RNA-seq analysis of control and *Isl1-Esr1*KO mouse uterus, we provided candidates for such paracrine factors that fulfill the following two conditions for stromal ESR1-regulated genes. These are 1) “genes that are upregulated by E2 in control mouse uterus” and 2) “highly expressed genes in E2-treated controls compared with E2-treated *Isl1-Esr1*KO mouse uterus”. We found several secreted growth factors and related genes, including *Igf1*, that fulfill these criteria. Fibroblast growth factors (FGFs) are expressed in the stroma in the presence of E2 and activate FGF receptor signaling in the uterine epithelium in a paracrine manner, leading to subsequent cell proliferation via MAPK activation^29^. Wnts play multiple roles in uterine physiology and diseases^30^ and contribute to stem cell-like characteristics in the uterus^31^. Several Tgfβ superfamily member genes were identified as stromal ESR1-mediating secreted factors where they were suggested to be possible regulators of cell proliferation and differentiation in the uterus during pregnancy and carcinogenesis^32–34^.

Regulation of cell proliferation in the uterine epithelium and stroma is important for implantation, and the establishment and maintenance of pregnancy. An important mechanism underlying this response is mediated by the expression of PGR and CEBPB, which could regulate cell proliferation in the stroma^20,35^. The *Isl1-Esr1*KO mouse uterus did not show stromal expression of PGR and CEBPB. In normal mice, stromal cell proliferation is independent of ESR1 expression, suggesting paracrine or juxtacrine regulation of stromal cell proliferation (Fig. S4). Therefore, it remains unknown whether regulation of *Pgr* and *Cebpb* gene expression is directly mediated through ESR1 in the uterine stroma. Epithelial expression of PGR and CEBPB is differentially regulated by estrogens. In control mice, CEBPB was induced by estrogens in both epithelium and stroma. By contrast, PGR was expressed in epithelial cells in the absence of E2 while E2 administration down-regulated PGR expression. In the *Isl1-Esr1*KO mouse uterus, epithelial CEBPB expression was probably mediated by epithelial ESR1 while epithelial downregulation of PGR failed to occur, although the mediating factor(s) remain to be elucidated. We were unable to evaluate whether implantation could be successful in the *Isl1-Esr1*KO model due to anovulation, which was likely due to deletion of ESR1 in ovary and/or hypothalamus-pituitary axis.

LTF is an epithelial secreted protein and a primary marker for estrogen actions in mouse uterine epithelium. Previous tissue recombination experiments suggested that both stromal and epithelial ESR1 were required for the production of E2-dependent epithelial LTF^9^. The current results supported this conclusion and the idea that overall estrogen action in the uterus is via stromal ESR1. Intriguingly, *Ltf* expression was augmented in the *Isl1-Esr1*KO OVX mice compared to OVX controls. Additionally, Mucin 1 (MUC1), also regulated by estrogen and secreted at the epithelial cell surface^36^, was increased in *Isl1-Esr1*KO mice compared to controls in the absence of estrogen (Table S5). Therefore, epithelial cells in *Isl1-Esr1*KO exhibit secretory characteristics normally seen after estrogen stimulation by stromal *Esr1* deletion.

### Gene expression in response to E2 in the Isl1-Esr1KO mouse uterus

The current RNA-seq analysis revealed that DEGs elicited by E2 at 6 h were decreased in the *Isl1-Esr1*KO mice compared with those of controls. This indicated that the majority of transcripts induced by E2 in the mouse uterus through ESR1 occurred in the stroma rather than the epithelium. This is consistent with previous RNA-seq analyses conducted using epithelial cell-specific *Esr1*KO mouse uterus in the early phase of estrogenic response^37^. We also evaluated gene expression with qRT-PCR analysis, and showed that expression of early estrogen responsive genes, *Lif* and *Aqp5* was upregulated at 2 h after E2 administration in both control and *Isl1-Esr1*KO mouse uterus. Expression of *Lif* and *aqp* genes were not increased in epithelial *Esr1*KO mouse uterus^10^; therefore, these genes are probably induced directly by the luminal and glandular epithelial ESR1. On the other hand, some genes, such as *Cdkn1a*, were not induced in either epithelial-specific or stromal-specific *Esr1* KO mice^10^ and the current study.

GO analysis was performed on the stromal ESR1-induced genes. In addition to cell proliferation-related genes, we found that “lipid metabolism” was one of the most enriched biological process terms. Most genes were biased in the E2-treated control group. Thus, stromal ESR1 contributes to metabolic regulation, which is definitely required for subsequent uterine physiological events during the very early phase of estrogen stimulation. Lipid metabolism is intriguing because a conditional deletion of *Ctnnb1/β-catenin* in mouse uterus transformed myometrial cells to adipocytes^38^. CTNNB1 is an effector molecule for Wnt signaling, suggesting that metabolic regulation by an ESR1-Wnt axis maybe important for tissue homeostasis in the uterus. Furthermore, treatment with E2 and the peroxisome proliferator activated receptor gamma (PPARγ)-specific agonist, rosiglitazone, induced abnormal uterine glands and atypical endometrial hyperplasia^39^. The direct PPARγ target gene, fatty acid-binding protein 4 (*Fabp4*) is expressed in the epithelium and is involved in embryonic implantation^40^. Therefore, stromal ESR1 regulates a variety of physiological events in the uterus, in part through regulation of lipid metabolism-related genes.

In the human uterus, endometrial cell proliferation is controlled by estrogen levels in the body during the menstrual cycle. Nevertheless, the mechanisms of cell proliferation at the tissue level in the normal uterine epithelium are still not well understood. Estrogen is strongly associated with the development of cancers and thus aberrant regulation of uterine cell homeostasis is involved in endometrial cancer and infertility. In this study, we used mice in which *Esr1* was knocked out in the entire uterine stroma to elucidate estrogen-mediated tissue interactions and regulation of estrogen actions. Overall, an improved understanding of the distinct roles of epithelial and stromal ESR1 will shed light on the mechanisms of estrogen-mediated homeostasis underlying disorders in female reproductive organs.

## Methods

### Mouse and treatment

C57BL/6J (Sankyo, Tokyo, Japan), *Isl1-Cr*e^41^, *Esr1*-null and *Esr1-floxed*^1^ mice were maintained under 12 h light/12 h dark at 23–25 °C, and fed laboratory chow (MR Standard; Sankyo) and tap water ad libitum. To obtain uterine stromal cell-specific *Esr1KO* mice (*Isl1Cre/* + *;Esr1*^*flox/*−^), *Isl1Cre/* + *;Esr1*^+/−^ male were crossed with *Esr1*^*flox/flox*^ female mice. For control mice, *Cre*-negative-*Esr1*^*flox/*+^ siblings were used. In most experiments, mice were ovariectomized under combination of anesthetic with midazolam (0.3 mg/kg body weight), medetomidine (4 mg/kg body weight) and butorphanol (5 mg/kg body weight) at 6 weeks of age and sacrificed at 8 weeks of age. For examining effects of estrogen, a single injection of 100 ng E2 (Sigma, St. Louis, MO, USA) was given to OVX mice and sacrificed 2, 6, 12, and 24 h after the injection. Some mice were given a single daily injection of 100 ng E2 for 3 days and sacrificed 24 h after the last injection. For examining effects of progesterone, OVX mice were primed with 100 ng E2 for 2 days. After resting for another 2 days, four daily injections of 1 mg progesterone (Sigma) with one injection of 50 ng E2 with the last injection of P4 on the fourth day.

All experiments involving animals and their care were conducted in compliance with ARRIVE guidelines. All experiments were performed in accordance with relevant guidelines and regulations. The present study was approved by the Animal Care and Use Committee at the Tokyo University of Science (No. K19013, K20013, K21011, K22012).

### Histology and immunohistochemistry

Hematoxylin and eosin staining and immunohistochemistry were performed as previously described^13,42^. For immunohistochemistry, paraformaldehyde-fixed, paraffin-embedded sections were incubated with the following primary antibodies: ESR1 (sc-8005), PGR (sc-538), CEBPB (sc-150), FOXA2 (sc-6554), αSMA (sc-53142, Santa Cruz, Santa Cruz, CA, USA). The sections were stained with the Vectastain ABC Kit (Vector Laboratories, Burlingame, CA, USA). Immunofluorescence analysis was performed with Alexa Fluor protein-conjugated secondary antibodies (Thermo Fisher Scientific, Waltham, MA, USA) and counterstained with Hoechst 33342 (Sigma).

For EdU-immunostaining, mice were injected with EdU at 50 mg/kg body weight. One hour after the injection, animals were euthanized and tissues collected. EdU-incorporated cells were detected using Click-iT EdU Imaging Kits (Themo Fisher Scientific) as described in the manufacture’s protocol. In some samples, ESR1 (sc-8005) was detected with Alexa Fluor protein-conjugated secondary antibodies (Thermo Fisher Scientific) and immunofluorescent imaging. More than 3 animals were analyzed, and representative pictures are shown.

### Quantitative reverse transcription-polymerase chain reaction (qRT-PCR)

Total RNA was isolated from each group using ISOGEN II reagent (Nippon Gene, Tokyo, Japan) then reverse transcribed with PrimeScript RT reagent Kit (Takara, Kusatsu, Japan). qRT-PCR was performed with a StepOnePlus Real-Time PCR system (Thermo fisher Scientific) with TB Green Premix Ex Taq II (Takara). The reaction profile consisted of 2 min 50 °C and 5 min at 95 °C followed by 40 cycles at 95 °C for 15 s and 60 °C for 1 min. The expression levels of the target genes were normalized against the expression level of the ribosomal protein L7 (*Rpl7*). Sequences of the specific primers are given in Supplemental Table S1. At least three samples were run in triplicate to determine sample reproducibility. A two-way ANOVA followed by a Tukey–Kramer test was used to analyze differences in gene expression. *p* < 0.05 was considered as significantly different.

### RNA sequence (RNA-seq)

RNA was extracted from the uteri of three mice to make one sample and three biological replicates (n = 3) from each group were analyzed. However, due to quality issues during the data analysis, control E2 and *Isl1-Esr1*KO E2 group were analyzed with N = 2. Total RNA was isolated from whole uteri using the ISOGEN II reagent and purified with RNeasy micro kit (Qiagen, Hilden, Germany) according to the manufacturer’s instructions. Total RNAs extracted from the uteri of three mice were combined into one sample, and three samples from each group were processed for RNAseq analysis at Macrogen Japan (Tokyo, Japan) using the NovaSeq6000 platform with the Truseq stranded mRNA library constructed for paired-end 100 bp applications, according to Macrogen’s protocol. The quality of output sequences was inspected using the FastQC program (version 0.11.2, available online at: http://www.bioinformatics.babraham.ac.uk/projects/fastqc). The reads from each biological replicate were mapped to the mouse genome (GRCm38.p6) for quantification by Salmon (version 1.2.1). Differentially expressed genes (DEGs) were calculated using DESeq2 package (version 1.22.2) in the SARTools package (version 1.6.6)^43^ with R (version 3.5.3)^44^. Gene ontology enrichment analyses were conducted using DAVID web service (version 6.8)^45^.

## Supplementary Information

Supplementary Tables.

Supplementary Figures.

## Data Availability

Sequencing data have been deposited in DDBJ under the accession code DRA016091 (https://ddbj.nig.ac.jp/search?query=%22DRA016091%22).
