# Supplementary material for: The role of mesenchymal estrogen receptor 1 in mouse uterus in response to estrogen
Source: Sci Rep. 2023 Jul 29;13:12293. doi: 10.1038/s41598-023-39474-y (PMC10387046; doi:10.1038/s41598-023-39474-y)
Supplement: Supplementary file 2 — Supplementary Figures. [file 41598_2023_39474_MOESM2_ESM.pdf]

Supplementary Figures

The role of mesenchymal estrogen receptor 1 in mouse uterus in response to estrogen

Keita Furuminato[1], Saki Minatoya[1], Eriko Seno[1], Tatsuki Goto[1], Sho Yamazaki[1], Moeka Sakaguchi[1], Kenji Toyota[1,2], Taisen Iguchi[3], Shinichi Miyagawa[1,*]

[1]Department of Biological Science and Technology, Faculty of Advanced Engineering, Tokyo University of Science, Katsushika, Tokyo 125-8585, Japan
[2]Noto Marine Laboratory, Institute of Nature and Environmental Technology, Kanazawa University, Noto, Ishikawa 927-0552, Japan
[3]Graduate School of Nanobioscience, Yokohama City University, Yokohama, Kanagawa 236-0027, Japan

*Correspondence:
Shinichi Miyagawa
E-mail: miyagawa@rs.tus.ac.jp
Phone +81-3-5876-1466

Supplementary Figure S1--2
Enlarged images for Figs. 1C, 1D, 2C, and 2D.

Supplementary Figure S2--3
ESR1 protein expression in the uterus of control and Isl1-Esr1KO mice at 3, 10, and 21 days of age.

Supplementary Figure S3--4
Ovarian histology of control, conventional *Esr1*KO, and *Isl1-Esr1*KO mice at 8 weeks of age.

Supplementary Figure S4--5
Diameter and the number of uterine gland in control and *Isl1-Esr1*KO mouse uterus.

Supplementary Figure S5--6
Immunofluorescent imaging of EdU-incorporating and ESR1 expressed cells.

Supplementary Figure S6--7
Cdkn1 gene expression at 2, 6 h, and 24 h after E2 administration.

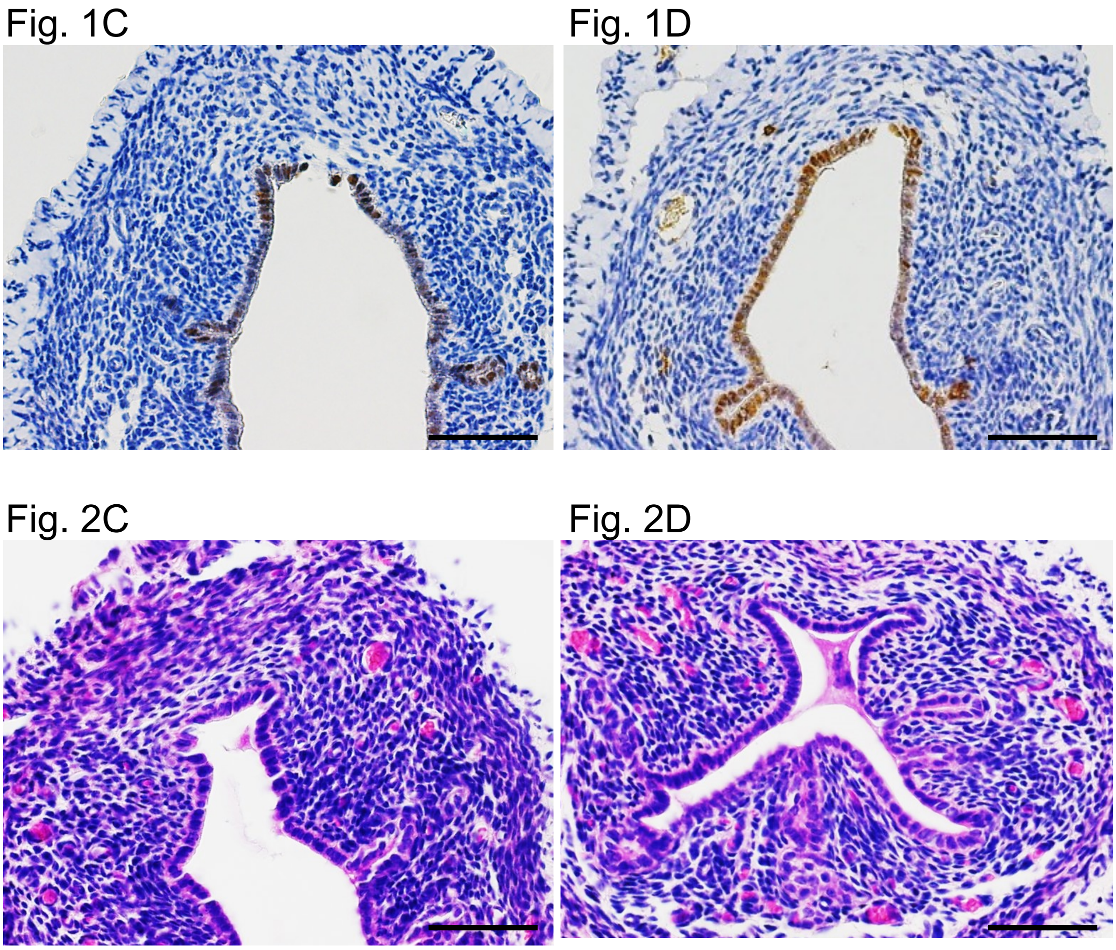

Fig. S1

Enlarged images for Figs. 1C, 1D, 2C, and 2D Scale bars, 50 μm.

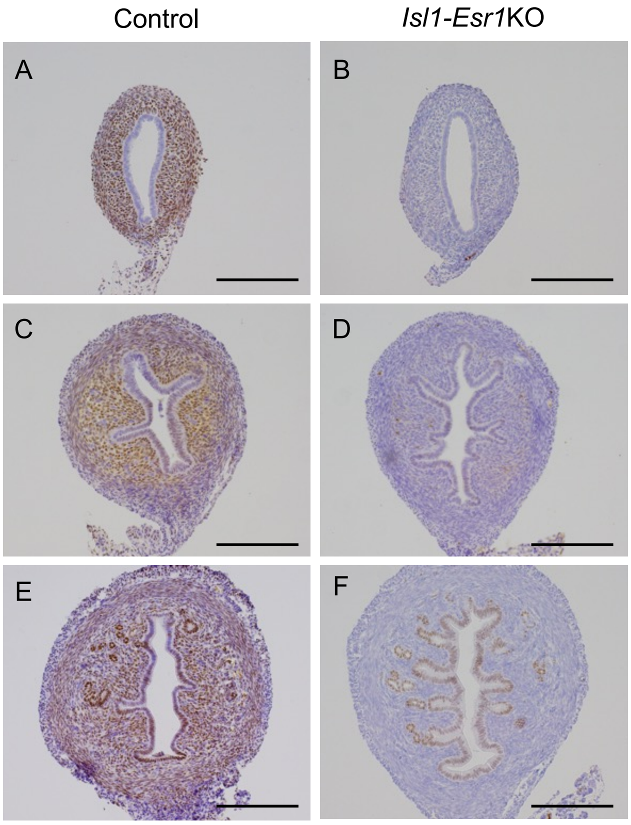

Fig. S2

ESR1 protein expression in the uterus of control (A, C, D) and *Isl1-Esr1*KO mice (B, D, F) at 3 (A, B), 10 (C, D), and 21 (E, F) days of age. Stromal ESR1 is lost at the neonatal period in the *Isl1-Esr1*KO mouse uterus. Scale bars, 100 μm.

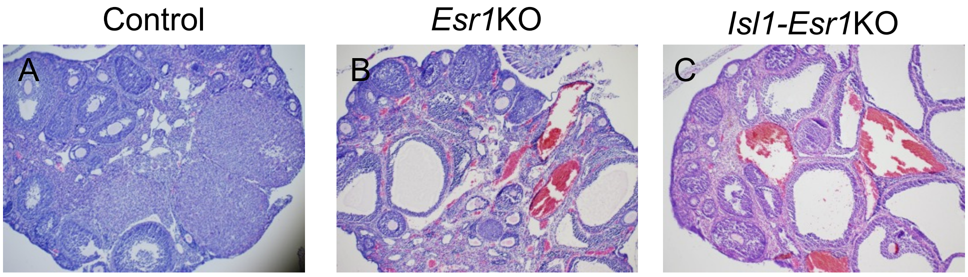

Fig. S3
Ovarian histology of control (A), conventional Esr1KO (B), and Isl1-Esr1KO mice (C) at 8 weeks of age. Intact mice exhibit mature follicles and corpora lutea. The ovary of Isl1-Esr1KO mice lacked corpora lutea, and accompanied by hemorrhagic cysts like that of conventional Esr1KO mice.

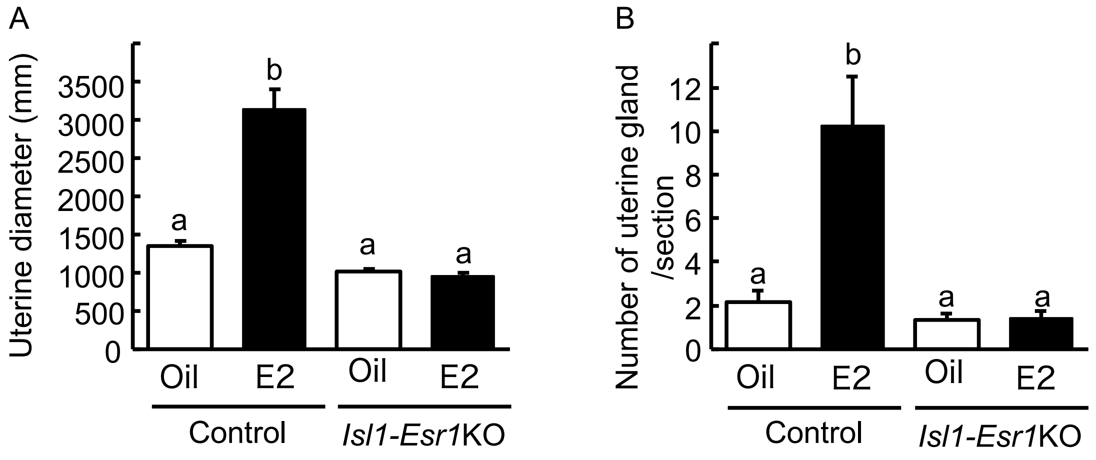

Fig. S4

Diameter (A) and the number of uterine gland (per section) (B) in control and Esr1KO mice. More than 5 animals were analyzed. Error bars represent SEM. * indicates significant difference compared with OVX group assessed by student's t-test ($p<0.05$)

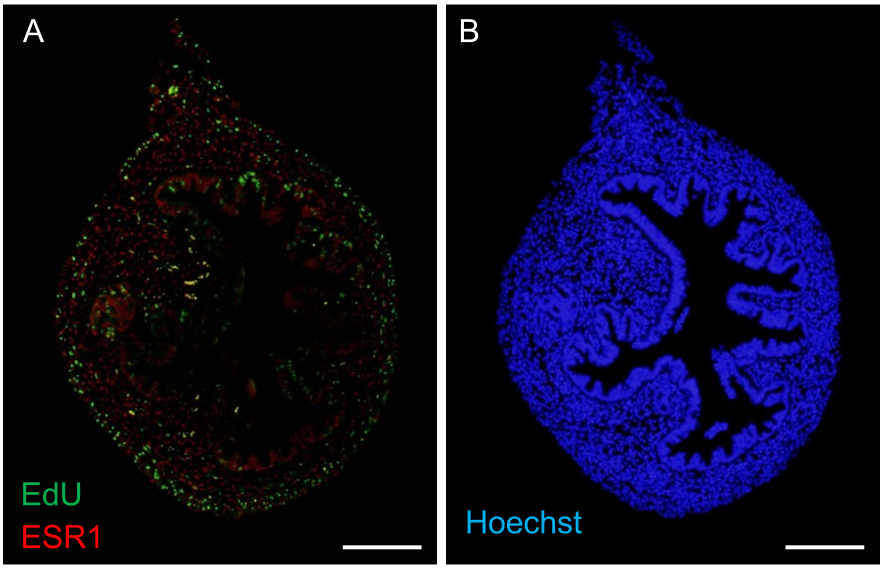

Fig. S5
EdU-incorporating cells (green) do not always overlapped with ESR1 expressed cells (red) in the wild-type mice uterus (A). Blue fluorescent signal (B) indicates Hoescht staining in the same image of EdU staining. Scale bars, 200 μm

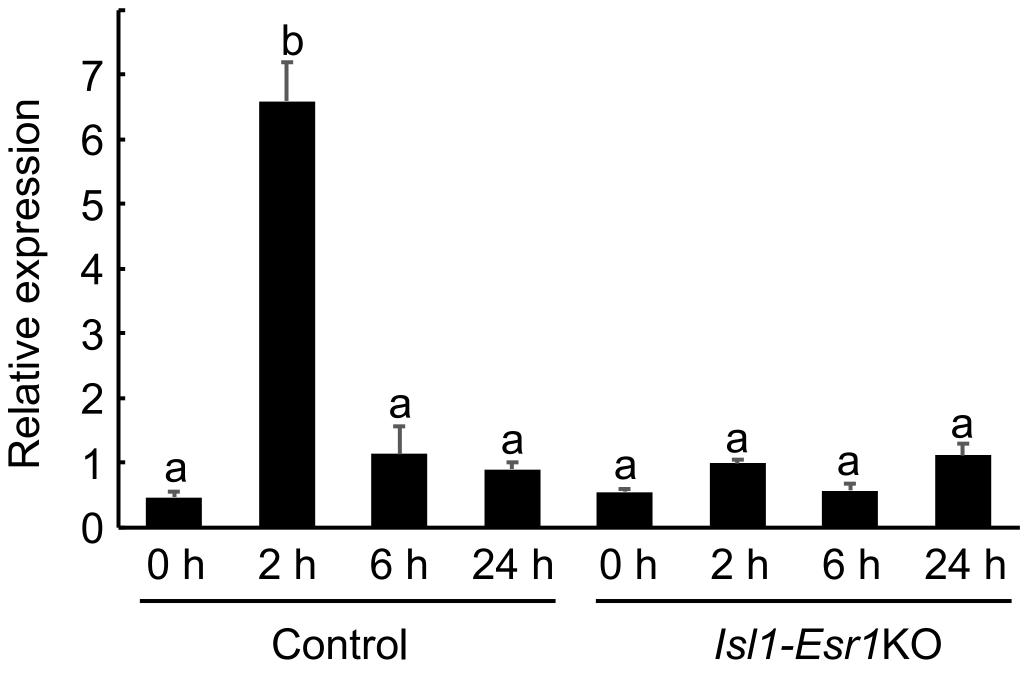

Fig. S6
Cdkn1 gene expression at 2, 6 h, and 24 h after E2 administration. Results are mean ±SEM. A two-way ANOVA followed by a Tukey–Kramer test was used and $p<0.05$ was considered as significantly different.
